# Characteristics of the suicidal soldier in the Israeli Defense Force-a review of literature

**DOI:** 10.1186/2054-314X-1-10

**Published:** 2015-04-06

**Authors:** Leah Shelef, Lucian Laur, Eyal Fruchter

**Affiliations:** 1 Psychology Branch, Israeli Air Force, Kragujevac, Israel; 2 Mental Health Department, Israel Defense Force Medical Corps, Ramat Gan, Israel

**Keywords:** Suicide, Israeli soldiers, Suicidality, Military stress, Adolescents

## Abstract

Suicide is the third leading cause of death among adolescents in most Western world countries. Similar findings have been reported among adolescents in Israel (including the Israeli army) in times of peace; nonetheless, suicide rate has decreased significantly in recent years.

In Israel, IDF service is mandatory and adolescents are obligated to serve by law. Therefore, the IDF is responsible under state and moral law to care for the physical and mental health of its soldiers. Additionally, there is an understanding that the Israeli soldiers represent a mentally healthy population as prior to their enlistment they undergo a series of tests and evaluations to determine their suitability for service. The IDF is one of the few organizations in the world that is comprised of the majority of a country’s healthy adolescent population. International literature defines this population (i.e., the adolescent population) as having the highest risk of suicidality. Moreover, the risk of suicide increases in the face of two additional circumstances within the context of military service: the army service as a stressor and the availability of weapons.

The IDF invests significant resources in delineating the characteristics of suicidal soldiers, realizing its importance for suicide prevention during military service. This article reviews studies regarding complete suicide cases of Israeli soldiers in the aim of characterising a ‘suicidal soldier’s profile’ to inform better screening and prevention policies.

## Introduction

The fundamental question of what suicide is, has been dealt with by many researchers, suggesting various view points for the phenomenon. Lethality of method and intent of the suicide act are very important in the assessment [1]. Thus, the definition of ‘suicide’ as a concept is not homogenous, and the differences begin with the attempt to explain what a “suicide act” is. The difference originates from the fact that the phenomenon is comprised of a broad range of behaviors, stemming from different motives, driven by different intentions and culminating in a broad array of results – ranging from Non Suicidal Self Injury to death. The innovative definition of suicide act refers even to the lethal potential of a suicide intention, even if there was no actual self-injury[1].

Suicide is the leading cause of death in most armies during times of peace [2, 3] including the IDF [4, 5]. As such, it is one of the issues being dealt with by military mental health professionals. The IDF is the only organization in the world, which encompasses within it, the majority of the country’s adolescent population [6]. This age- group is defined as having the highest risk of suicidality [7]. The risk increases when adding the army service as a stressor [8], and the availability of weapons [4]. The three parameters together enhance the suicidality risk within the IDF significantly [4, 6].

It has been found that the suicidal soldier’s characteristics tend to be male, aged 19–20, with an intact medical profile, with high intellectual capabilities, higher than average motivation and psychometric abilities and front-line combat unit posting [9]. He is typically in his first year of military service and the suicide act occurs most frequently during December, and least frequently in October [10]. It has also been found that most suicides occur on a Sunday which is the first work day following the weekend in Israel [11]. Suicide is a rare event, yet every act has large impact on the unit, family and friend. Hence the IDF makes great efforts in trying to characterize the suicidal soldiers, realizing it is critical to identify individuals at higher risk of suicide during their military service. This article will review most studies regarding competed suicide cases of Israeli soldiers in order to form a character profile for the suicidal soldier, and enable better screening and prevention.

## Review

### Gender differences

A record study that looked at soldiers who committed suicide between the years 1974–2001, including soldiers between the ages of 18–51 (mandatory service , professional and reserve soldiers), found that of the 830 soldiers who had completed suicide, 60% were young males [8, 11].

One of the reasons for the lack of research on military gender and suicidality is the low suicide rates of female soldiers in comparison to those of males. This finding is also found in general population studies [7]. Service in the IDF in Israel is mandatory for males and females; however, in reality, only 40% of females enlist in comparison to 60% of males. Therefore, the percentage of female recruits is lower than that of the males even though those who do recruit have higher quality screening scores in comparison to those of the males [12]. Moreover, males serve for longer periods – 3 years in comparison to two years mandatory service for female [6]. There is also a difference between male and female soldiers when comparing their basic training and their assignment into military professions. The length of basic training for females is shorter than for males, and most of the profession training periods for females are shorter [6]. Additionally, even though more combat roles are opening up for females, they can volunteer for a combat role; whereas for males, these appointments are compulsory [12].

### Medical profile

All young Jewish individuals and Druze males, who turn 17 years of age, are carefully screened for physical and mental pathology prior to military induction, in order to determine their eligibility for service [5, 6]. This screening is based on a physical and psychiatric evaluation in which draftees are assessed by professional and paraprofessional interviewers who screen the entire population of recruits [13]. It is important to identify difficulties or problems among the recruits so that their medical profiles will suit their military assignment [5]. The profile determines the classification of the soldier to various units, including to combat and non-combat [13].

The medical profile is a non-sequential scale ranging from 97 as most physically capable to 21/24 for those found to have a major and debilitating disease – physical or mental - who are then waived from mandatory service. The medical profile can be changed during military service either by the authorities or by the soldier as a consequence of changes in the soldier’s physical or mental state [13].

Before 2008 the mental health indicators within the medical fitness profile reflected the individual’s functional level for the military rather than a precise clinical diagnosis as in physical illnesses [5] since 2008 on, the mental health parts are based on the ICD- 10 [14]. A study conducted over an eight-year period (1990–1998) showed that soldiers with a mental health profile indicator constituted 8.9% ± 1.2% of the entire IDF population and 13.1% ± 4.8% of those who completed suicide [5].

In their study “Death without Warning,” Apter and his colleagues [8] performed psychological- psychiatric autopsies on 43 suicide cases of individuals between the ages of 18–21 who committed suicide during their mandatory military service. They found that 48% of the soldiers were characterized as having some difficulties under pressure. Different stressors were found to be related to suicide: romantic rejection (30%), and financial and familial problems (33%). Only among 28% was stress actually related to the military service. In addition, the findings showed that 53.5% of those who committed suicide seemed to have suffered from depression that emerged very close to the time of the suicide itself. Seven percent suffered from adjustment problems, seven from schizophrenia, 4.7% from dysthymia, 2.3% from an obsessive compulsive disorder, 2.3% from minor mental retardation, and 18.6% had no diagnosed disorder [8]. The attempt to diagnose personality types showed that 37.2% of the individuals were of schizoid personality, 23.3% of narcissistic personality, 7% of an avoiding personality, 4.7% of borderline personality, 14% of other personality types, and among 9.3% no personality diagnosis was found [8].

A study examined six different groups of soldiers who expressed some degree of suicidal behavior in an attempt to find similarities and differences between the groups. A difference was found between those subjects that executed a severe suicide attempt, and the soldiers that carried out a non-severe attempt or merely threatened with suicide but did not cause any self-harm. The subjects who executed a severe suicide attempt, expressed some form of psychiatric mental illness, mainly major depression, in close proximity to the suicidal episode. Although symptoms of depression and other psychiatric mental illness could also be observed in the other groups, there were more psychiatric diagnoses of adjustment disorders and almost no diagnoses of major depression were found [9].

Finally an autopsy report study which examined cases of soldiers who had completed suicide during their military service, found that among most soldiers, in the three weeks prior to the suicide, there was no change in their military functioning. 83% of the soldiers who completed suicide did exhibit signs of distress, but their military functioning was almost not effected [15].

A recent retrospective nested case–control study of all active-duty Israeli military male personnel between 2002 and 2012, supports the findings indicating the difficulties in recognizing signs of distress in the period preceding suicide. This research included 170 completed suicides and 500 matched controls. The results indicate that whereas 38.3% (n = 62) of suicide cases contacted a primary care physician within the last month before death, only 27.6% (n = 27) of suicide cases contacted a mental health specialist during their entire service time. The primary care physician contact rate within one month before death or in death day did not differ between suicide cases and matched controls (38.3% (n = 62) vs. 33.8% (n = 161), χ2 = 1.05, df = 1, p = 0.3). Furthermore, no difference was found in rates of last contact with the primary care physician within the last week, three months or six months before death between groups. More suicide cases contacted a mental health specialist within the service time than matched controls (27.6% (n = 27) vs. 13.6% (n = 50), χ^2^ = 10.85, df = 1,p = 0.001). Even though the primary care physician contact rate by military suicides is slightly lower than that reported for civilian suicides prior to their death (38% versus 45%), it is higher than mental health specialist contact rate and higher than civilian suicides in the same age range [16].

The meaning of this finding is that even if major depression was evident in the weeks before the suicide act, it wouldn’t be at its highest intensity, thus would not be observed as dysfunction, nor during first physician visits [16]. Rather, the major depression would only be evident in retrospect - after the completed suicide [15].

### Military seniority

The beginning of military service, including basic training and the profession training, is a critical adaptation period [8]. The adolescence is introduced to a new situation requiring high coping capability whether the new situation is subjectively perceived as positive or negative. During this period, the recruit deals with a number of changes simultaneously: changes in living conditions, residence change and separation from familiar support systems, changes in sleeping and eating conditions - at times with a deprivation in these basic needs. The recruit faces changes in social activities and coercion to form relations with others that were not selected in free will as friends, dealing with responsibility, coping with a new profession, using weapons, and dealing with hierarchal commanding authorities [5, 6]. All these changes and difficulties contribute to the fact that a high percentage (38%) of the suicides in the IDF occurred within the first six months of service, especially during basic training and the initial profession training [8, 17].

A research study that examined 404 soldiers who completed suicide between the years 1974–1994 found that 56% of them committed suicide during the first year, while26% and 18% completed suicide during the second and the third years respectively (Borkov, C: Israel Defense Forces suicides between 94-74, Research Report, IDF Press (Unpublished)), and 10% completed suicide in the last month prior to being discharged from military service (Borkov, C: Israel Defense Forces suicides between 94-74, Research Report, IDF Press (Unpublished)).

One other explanation for the occurrence of suicidal behaviors in the first six months of service is related to self-identity formation- meaning the psychological way one perceives himself (meaningful or not, succeeding or not etc.). A study conducted in the IDF among 32 soldiers that had made serious suicide attempts with intent to die, soldiers that had been diagnosed as suffering from emotional distress without suicide ideation or a history of previous suicide attempts, and a control group, found that self-identity is decreased during basic training and initial profession training. Likewise, it was found that military stress begins upon recruitment, and suicidal tendencies reach their peak in the midst of the basic training period. Military stress begins to subside only in the period between mid-basic training and the end of the initial profession training [18].

### Quality data

As described above, prior to induction, IDF recruits undergo intensive screening procedures. One of the screening tasks refers to determining quality potential of the recruit by passing him through psychometric evaluation, a semi-conducted coping skills interview and screening for past adaptation difficulties [5, 6, 8]. At the end of this evaluation, the recruit gets an intellectual evaluation score parallel to the IQ scale and a quality potential scale [8].

#### Intellectual capabilities

The psychometric evaluation includes cognitive testing of scholastic abilities and personality measures targeted at assessing combat suitability and future military performance [13].

In one study of suicides between the years 1974–1994, no significant differences were found between the intellectual capabilities of those who completed suicide compared to the general recruit population (Borkov, C: Israel Defense Forces suicides between 94-74, Research Report, IDF Press (Unpublished)). A smaller study that examined 43 cases of soldiers in compulsory military service who committed suicide during the mid-1980’s found that those who had completed suicide had significant higher intellectual scores then those in the general population [8]. In another study that was conducted between the years 1983–1988, the authors compared three groups: a group of soldiers who completed suicide, a group of soldiers who attempted suicide using fire arms, and a group of soldiers who attempted suicide using other means. The authors found that recruits with higher quality screening scores had a significantly higher suicide risk. Lower quality screening scores were found to be related to higher suicide attempt risk [8, 9].

#### Adaptation difficulties

Recruits who are identified as having adaptation difficulties in the first interview, may receive special evaluation by MHO (mental health officers) in the recruitment centers. The IDF has defined categories for “less resilient” (as defined by the term *‘* special-needs’ [5] recruits who may be assigned to less stressful service environments and occupations, receive different initial training, or receive enhanced support during their service [5].

One particular study investigated three groups. The first group included a problematic soldier population (termed MAKAM), which is characterized as having some emotional and behavioral problems. The second group was that of soldiers with a recognized moderate mental disorder due to one or more psychopathology. The third group was characterized by having functional difficulties which made it harder for them to adjust to service. The study took place over a nine year period (1990–1998), and found that there was a significant decrease in suicide rates among the first group population as a result of focused treatment and support provided to them by more attentive commanders and MHO throughout their military service. This is an important finding due to the fact that this sub-group of soldiers included soldiers with a low education level, un-met psychological needs, a history of delinquency and criminal records, emotional and behavioral problems, a low frustration threshold, low abilities for forming interpersonal relationships, difficulties with problem solving, and trouble with accepting authority and responsibility. These prominent characterizations are identified in research literature as significantly increasing suicide risk. Despite this, the suicide rate among this first group was the lowest. The highest suicide rates were found among soldiers defined as having adjustment difficulties [5]. This last finding was supported by another study that examined 181 cases of soldiers who completed suicide from the beginning of 1989 to the end of 1994 (Borkov, C: Israel Defense Forces suicides between 94-74, Research Report, IDF Press (Unpublished)). It is unfortunate that data collected on the years 2000–2003 shows that 80% of those who committed suicide in the IDF did not turn to mental health professionals for help [12], (Borkov, C: Israel Defense Forces suicides between 94-74, Research Report, IDF Press (Unpublished))], because from the “MAKAM” study it is evident that when soldiers in need gets the right attention and help from commanders and MHO, they will have a good chance to complete the army service unharmed [5].

### Military service

One study, conducted between the years 1983–1988, compared three groups: A group of soldiers who committed suicide, a group of soldiers who attempted suicide using fire arms, and a group of soldiers who attempted suicide using other means. The researchers of this study found that while among the soldiers who committed suicide there was an over representation of combat soldiers, among the soldiers who attempted suicide there was an over representation of non- combat soldiers [19]. In other words, completed suicides and near-fatal suicides were more likely to occur in stressful front-line combat units [9, 17].

Another study compared a population of combat and non-combat soldiers that completed suicide (N = 429) with two matching groups: combat and non- combat soldiers with no history of suicide attempts (N = 299). Results from this study indicated that the soldiers from the two groups that completed suicide showed higher adaptation capabilities and high motivation to serve in the military, in comparison to the two groups of soldiers with no suicide attempt history. Nonetheless, differences were found between soldiers from combat and non- combat units who completed suicide. The combat population with higher motivation to service showed a higher degree of independence, and had far fewer unit and military assignment changes, in comparison to the soldiers from the non- combative units who committed suicide [13].

This finding could be perceived as contradicting the findings mentioned above, which indicate that the population characterized as “less resilient”, that is to say those with adjustment problems, had a higher likelihood of suicide [5]. In the present study, the suposingly “most resilient” population is combat soldiers with higher intelligence capability, yet they have a higher risk for suicide [13]. The apparent contradiction is due to the differences between the two studied populations. The first study examined and compared a population that had been diagnosed by MHO (problematic soldier, psychopathology soldier, soldiers with adjustment difficulties). The second study population was an unknown population and was not diagnosed by MHO.

### Reasons for suicide

An analysis conducted in the IDF found six qualitative factors to be the most significant for the feeling of military stress: hard physical labor, long work hours, homesickness, negative life events (death of a family member, parental divorce, and sexual orientation), disagreement with authority – especially with commanding officers that are viewed as being unfair, and dissatisfaction with one’s placement. These factors are shared by soldiers in combat and in combat support units [20].

This finding was supported by another research study, stating higher subjective perception of military stress and self-identity formation (already mentioned), and its psychological effects as two main reasons contributing to military suicide [19].

Apter’s study, which examined 43 cases of soldiers that completed suicide found 48% of the soldiers were characterized as having some difficulties under pressure. 28% of them were directly related to military service, 30% were related to romantic rejection and 33% were related financial and familial problems [8].

An additional study which examined 181 military suicide cases identified 245 different reasons for suicide, although in many cases, no single reason was found (Borkov, C: Israel Defense Forces suicides between 94-74, Research Report, IDF Press (Unpublished)). The full number was not published, but the following were the most common reasons for suicide:

- Mental disorders and trauma
- Personal anxiety- emotional instability, fear of failure and concerns about discharge from the army.
- An immature personality - developmental and behavioral disorders and romantic difficulties.
- Adjustment disorders- social and functional adjustment difficulties to the military system.
- Mental burnout - loss of self-esteem, feelings of stress due to the military conditions.

Factor analysis indicated three areas of reasons for suicide: 41% fall under personal and emotional problems, 29% fall under relationship problems with family, and 30% fall under functional and adaptation difficulties in the army (Borkov, C: Israel Defense Forces suicides between 94-74, Research Report, IDF Press (Unpublished)). Enhanced differences were found in reasons for suicide while comparing combat and non-combat soldiers. The main reason for suicide among combat soldiers was loss of self-esteem, while the main reasons of non-combat soldiers were mental health and family problems (Borkov, C: Israel Defense Forces suicides between 94-74, Research Report, IDF Press (Unpublished)). In Israel there has only been one study that examined gender differences and suicidality during the military service. The study analyzed life stories of 50 male soldiers and 19 female soldiers who committed suicide during their military service [12]. When trying to pin-point the event which led to the suicidal crisis and trigger it, the researchers found that crisis pertaining to the military service was common (42% of the male soldiers and 63% of the female soldiers). The crisis situations included difficulties adjusting to the military system, to the military society, and to the harsh service conditions. They also found the following as influencing factors: disagreement with commanding officers, feelings of failure in completing the military mission, and disappointment with the military service and with the locationof service [12]. The study found that the emotional needs that were not fulfilled in the soldiers’ lives were similar among both sexes, except for the needs of acceptance and inclusion (higher for female soldiers) and the need for belonging (higher among the male soldiers). One of the explanations this study provides is related to the familial differences existing between the male and female soldiers [12]. The study showed that more female soldiers were raised in a harsh familial atmosphere, which included conflicting relationships, fighting, abuse and incest than male soldiers [12]. These findings are supported by another study that was conducted among soldiers who committed suicide and found that one third of the male soldiers and a half of the female soldiers had problematic familial relationships [8].

In addition, the study found that the suicide process is related to low self-esteem among both sexes, though more so in female then in male soldiers, probably contributing to the feelings of failing in the missions and not belonging. It was also found that females had a higher rate of depressive or anxious moods which worsened closer to the time of the suicide act in comparison to male soldiers [12]. self-disclosure of difficulties were also identified as higher among those who committed suicide, probably making it harder for these soldiers to ask for help and share difficulties. This was found in both sexes, but higher in the male group. Emotional regulation components and social and romantic relationships were found as elevating suicide rates for both males and females. Among male soldiers there was a higher frequency of suicides based on aggression and self-regulation difficulties in comparison to the female soldiers [12]. Furthermore, it was found that among both sexes there have been previous suicide attempts that preceded the actual suicide, although there was a difference with respect to the attitude towards death. While most of the male soldiers had a single suicide attempt, female soldiers made a number of suicide attempts before the actual suicide act took place [12].

## Discussion

This article reviewed most of studies conducted in the last 3 decades on suicide among IDF soldiers. All of the studies attempt to characterize the suicidal soldier as well to identify elements unique to the various stages of military service that may contribute to the risk of suicide. The studies condutcted on the IDF showed that the completed suicide acts were done by relatively more combat unit soldiers, and by soldiers who were highly intellectual, had high motivation, and were considered of high quality [9, 12, 13, 20]. They were also from families of higher socio-economic status, and usually didn’t have any prior suicide attempts [21]. Unfortunately, it is still hard to distinguish between the different suicide groups based on the data collected at induction to the army [22], so this effort should continue.

To date, there is broader knowledge within the IDF regarding the characteristics of the suicidal soldier (both completed and attempted suicide), as presented throughout this article. This knowledge includes- military assignment data, different population aspects, and causes and risk factors stemming from the different service periods. All this knowledge is shared with all MODs and commanders, and thus contributes to the success of the IDF suicide prevention program, which has reduced the military suicide rate in recent years by almost 42% [23].

Yet despite a strict filtering system, and the suicide prevention program, soldiers still commit suicide during their military service. Research and prevention programs receive priority in the IDF in general, and in the Military Mental Health Department within the Medical Corps in particular, in order to continue reducing the suicide rates.
